# Development and Characterization of Lightweight Geopolymer Composite Reinforced with Hybrid Carbon and Steel Fibers

**DOI:** 10.3390/ma14195741

**Published:** 2021-10-01

**Authors:** Agnieszka Baziak, Kinga Pławecka, Izabela Hager, Arnaud Castel, Kinga Korniejenko

**Affiliations:** 1 Chair of Materials Engineering, Faculty of Material Engineering and Physics, Cracow University of Technology, Jana Pawła II 37, 31-864 Cracow, Poland; agnieszka.baziak@student.pk.edu.pl (A.B.); kinga.plawecka@pk.edu.pl (K.P.); 2 Chair of Building Materials Engineering, Faculty of Civil Engineering, Cracow University of Technology, Warszawska 24, 31-155 Cracow, Poland; izbela.hager@pk.edu.pl; 3 School of Civil and Environmental Engineering, University of Technology Sydney (UTS), Sydney, NSW 2007, Australia; arnaud.castel@uts.edu.au

**Keywords:** geopolymer composite, lightweight composite, hybrid reinforcement, cenosphere, microsphere, steel fiber, carbon fiber

## Abstract

The aim of this paper is to analyze the influence of hybrid fiber reinforcement on the properties of a lightweight fly ash-based geopolymer. The matrix includes the ratio of fly ash and microspheres at 1:1. Carbon and steel fibers have been chosen due to their high mechanical properties as reinforcement. Short steel fibers (SFs) and/or carbon fibers (CFs) were used as reinforcement in the following proportions: 2.0% wt. CFs, 1.5% wt. CFs and 0.5% wt. SFs, 1.0% wt. CFs and 1.0% wt. SFs, 0.5% wt. CFs and 1.5% wt. SFs and 2.0% wt. SFs. Hybrid reinforcement of geopolymer composites was used to obtain optimal strength properties, i.e., compressive strength due to steel fiber and bending strength due to carbon fibers. Additionally, reference samples consisting of the geopolymer matrix material itself. After the production of geopolymer composites, their density was examined, and the structure (using scanning electron microscopy) and mechanical properties (i.e., bending and compressive strength) in relation to the type and amount of reinforcement. In addition, to determine the thermal insulation properties of the geopolymer matrix, its thermal conductivity coefficient was determined. The results show that the addition of fiber improved compressive and bending strength. The best compressive strength is obtained for a steel fiber-reinforced composite (2.0% wt.). The best bending strength is obtained for the hybrid reinforced composite: 1.5% wt. CFs and 0.5% wt. SFs. The geopolymer composite is characterized by low thermal conductivity (0.18–0.22 W/m ∙ K) at low density (0.89–0.93 g/cm^3^).

## 1. Introduction

Geopolymers are a class of synthetic inorganic aluminosilicate materials usually formed by the reaction of aluminosilicates (e.g., fly ash, metakaolin) with a silicate solution (e.g., Na_2_SiO_3_, K_2_SiO_3_) under strongly alkaline or acidic conditions (e.g., NaOH, KOH, H_3_PO_4_) [1,2]. Products based on geopolymers are characterized by very good properties such as compressive strength, thermal stability, acid resistance, and fire resistance or dimensional stability [2,3]. This gives the opportunity to use them in construction, to immobilize toxic waste and heavy metals (e.g., waste from asbestos, radioactive waste), or as refractory coatings in the aerospace industry [3,4]. However, the relatively low resistance to brittle cracking is a limitation for the use of these materials in many areas. For this reason, the topic of strengthening geopolymers using fibers is developing more and more dynamically [5,6].

The addition of fibers can improve the mechanical properties of the material obtained and change the nature of the fracture from brittle to more ductile, allowing new applications for the composite [7,8]. Reinforcing geopolymers with fibers gives the opportunity to improve bending strength and increases the amount of energy absorbed by the material before damage occurs. The addition of fibers reduces the number of cracks and their dimensions in the material, which in turn minimizes the damage caused by cracking and keeps the material cohesive for longer periods of time under the given load [8,9]. Such behavior is important, especially in emergency situations, such as fires or earthquakes, in which a person has more time to leave the endangered place. For this reason, reinforcing geopolymer matrices with fibers is a very interesting design solution that stands out from others currently available on the market [7,10,11].

There are various classifications of fibers, according to the methodology proposed by Ranjbar and Zhang, fibers used as reinforcement in geopolymer composites can be divided into five categories: metal, carbon, natural polymers, synthetic polymers, and other inorganic [12]. Depending on the type of fiber used, the mechanical, functional, or thermal stability of the material is different [12,13]. In addition, different types of fiber can be combined with each other to obtain the so-called hybrid material reinforcement. The use of different types of fiber in the right combination can effectively improve the mechanical properties of the geopolymer [2,7]. In the material obtained in this way, a synergistic effect appears, i.e., its resultant properties are higher than possible for each of the components separately [2,14]. In the provided research, steel and carbon fibers were selected as reinforcement due to their high potential to increase the strength properties of geopolymer composites [15,16].

The production of light building materials entails a number of benefits, such as a reduction of the load-bearing capacity of the structure, better thermal and acoustic insulation of buildings, and reduction of transport and assembly costs [17,18]. Structural elements made of them may have reduced cross-sectional dimensions and high durability, which allows for reducing the occupied storage space. Many lightweight concrete structures are built in seismically active areas. In this case, the advantages of using these materials are their low density, high fatigue strength, and resistance to dynamic loads [18,19]. Material density can be reduced by using lightweight and porous aggregates [19,20]. Concrete, including the geopolymer one, is considered light when its dry density is within the range of 800–2000 kg/m^3^ [20,21]. Examples of aggregates that can be used to obtain geopolymeric materials with relatively high strength and low density are microspheres [22,23,24,25,26,27], vermiculite [28,29], granulated foam glass [30,31,32], expanded polystyrene (Styrofoam) [29,33], pumice [34,35], perlite [36,37,38], and other expanded materials, e.g., clay or glass [39,40,41].

Microspheres, because of their anthropogenic origin and the fact that they are obtained as a by-product, are an interesting raw material that can be used in the production of light materials. They constitute a specific fraction of fly ashes produced as a result of burning solid fuels in pulverized furnaces. They are hollow spherical aluminosilicate particles with a density lower than that of water (0.2–0.8 g/cm^3^) [22,23]. Microspheres are formed at the stage of transformation of coal mineral substances during combustion, and their spherical shape is related to the cooling and solidification of ash particles around gases (i.e., CO_2_ N_2_, CO, O_2_, and H_2_O) [22,24]. The walls of the aluminosilicate grains are characterized by an amorphous structure with crystalline inclusions. There are two methods of obtaining microspheres from combustion waste: wet and dry. The wet method is based on the sedimentation phenomenon and, due to its simplicity, is used on an industrial scale. Unlike obtaining microspheres by the wet method, manufacturing them by the dry method is complicated, costly, and so far, not used on an industrial scale [22,23]. The content of microspheres in the ash depends on many factors, such as the properties of the combusted coal or the combustion technology used [24,25]. It is estimated that the particles constitute a negligible part of the furnace waste and their average content in ash is 1.1%. The chemical composition of the microspheres is similar to the composition of type F fly ash [25,26]. The main phases and minerals that build the microspheres are aluminosilicate, glass, mullite, quartz, calcium silicates, sulfate, calcite, and iron oxides. The solid phase of the microspheres is dominated by SiO_2_ at the level of 50–65%, Al_2_O_3_ at the level of 19–65%, and Fe_2_O_3_ at the level of 0.7–6.5%. The other relationships exist in negligible amounts [23,26]. The aluminosilicate grains have a diameter in the range of 125–500 µm, of which the largest fraction by volume are grains with a diameter between 250 and 300 µm. Microspheres are characterized by low open porosity of the walls (resulting in low water absorption and frost resistance) and a small surface area (<500 cm^2^/g). They also have high resistance to chemical factors, high and low temperatures, compression, and crushing resistance. The bulk density of the aluminosilicate grains shall not exceed 0.45 g/cm^3^. In addition, they are characterized by they have fire resistance and a low thermal conductivity coefficient, the values of which change slightly with increasing temperature. Additionally, the microspheres have the ability to dampen vibrations and do not have any harmful effects on living organisms. Due to their numerous and sought-after properties, microspheres can be used as a light filler, improving the thermal insulation properties of mortars and concretes [22,23,24,27].

The main objective of this article is to analyze the influence of hybrid fiber reinforcement on the properties of a lightweight, fly ash-based geopolymer. The topic of the article is related to the gaps identified in the literature. Geopolymers with hybrid reinforcement investigations are very limited, especially lightweight materials [2,7], and there is a lack of investigation for lightweight materials where microspheres were used as aggregate. The selection of microspheres as light aggregates was made on the basis of the literature analysis and taking into account environmental aspects. Both the fly ash and the microspheres are by-products of the combustion process, which made it possible to obtain an environmentally friendly material that complies with the philosophy of implementing a circular economy. Hybrid reinforcement of geopolymer composites was used to obtain the optimal strength properties, i.e., compressive strength due to steel fiber and bending strength due to carbon fibers. This composition has not yet been investigated. The reinforcement used could potentially expand the application area for lightweight geopolymers, especially in the aeronautic, automotive, and building industries.

## 2. Materials and Methods

### 2.1. Materials

**Figure 1:**
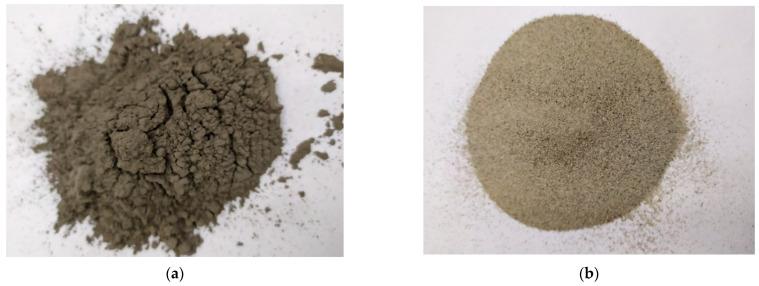
(**a**) Fly ash; (**b**) Microspheres.

The research was carried out on geopolymer composites with a matrix made of fly ash (FA) and microspheres–MS (TEAM-TRADE S.R.O., Prague, Czech Republic). Both fly ash and microspheres contain a large amount of aluminum and silicon, so they can be successfully used as matrix components of light geopolymer composites (Figure 1).

The fly ash used to carry out the empirical part of the work was supplied from the “CEZ Skawina” CHP plant (Skawina, Poland). This ash was in the form of fine dust with a gray color (Figure 1a). The oxygen composition of the ash is presented in Table 1 (information given by the producer).

**Figure 2:**
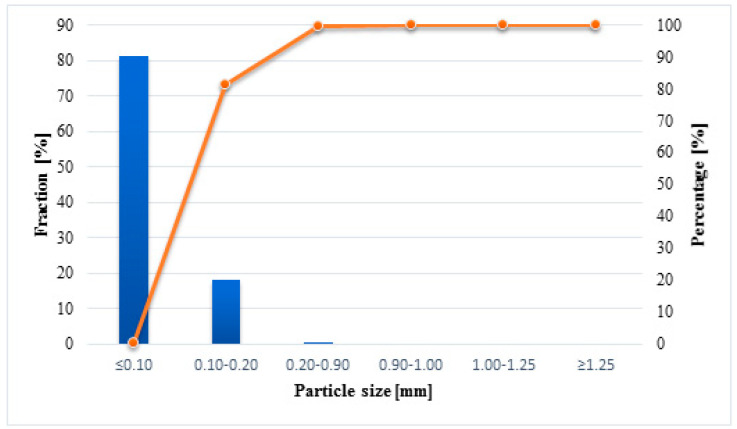
Particle size distribution histogram and cumulative particle size distribution curve.

FA has a typical composition for class F. It contains a large amount of Al_2_O_3_ and SiO_2_, which play an essential role in the geopolymerization process. In addition, it is located there is a small amount of CaO in it, which means that the geopolymerization process has a chance of a slower course. The composition of the ash also includes a large amount of iron in the form of hematite (5.767%), which may have a negative impact on the process of dissolving the ash grains. Additionally, the particle size analysis was performed using sieves. It allows the preparation of the distribution histogram with the particle size distribution cumulative curve for FA (Figure 2).

The FA used in the experimental part of the work has the highest share of the grain fraction with a particle size of <0.10 mm. There are no grain fractions with a particle size >0.9 mm at all.

**Figure 3:**
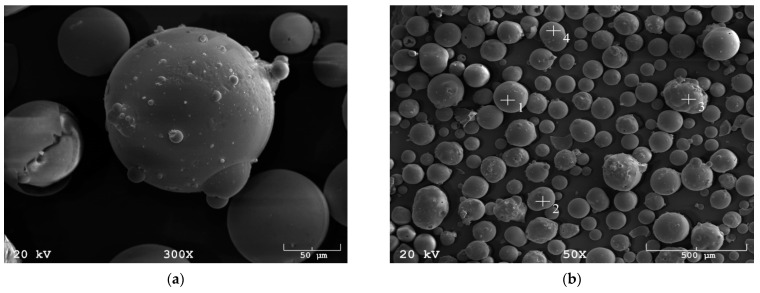
(**a**) SEM image: microspheres, magnification 300× (**b**) SEM image: microspheres, magnification 50×.

The microspheres (MS) were brown fine dust (Figure 1b). The information from the supplier was quite generic because for the MS supplied a microstructural investigation was provided using a JEOL JSM-820 scanning electron microscope EDS (IXR Inc., Austin, TX, USA). In the pictures taken with the scanning electron microscope (Figure 3), regular spheres with a size of about 130 μm can be seen.

**Figure 4:**
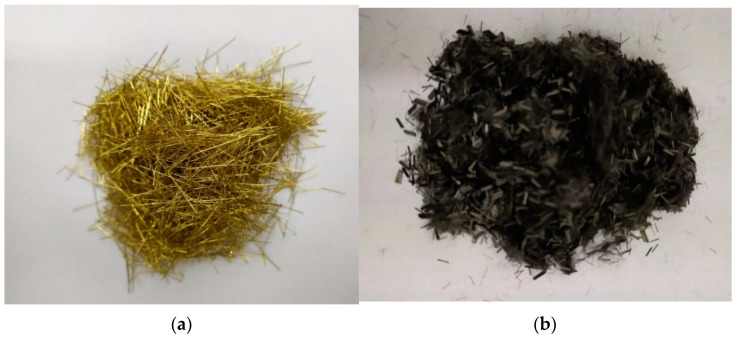
(**a**) Steel fibre; (**b**) Carbon fibre.

Two types of fibers were selected for the tests: steel fibers (LA MATASSINA S.R.L., Isola Vicentina, Italy) and carbon fibers (P.P.H.U. SURFPOL Jacek Woźniak, Rawa Mazowiecka, Poland)-Figure 4. Copper-coated steel fibers with a length of 20 mm and a diameter of 0.3 mm were used for the research. According to the producer, they were characterized by the following properties: breaking strength >1100 N and ultimate elongation <2% (Figure 4a). Short carbon fibers 3 mm long and 7 µm in diameter were used for the research. According to the supplier, they were characterized by the following properties: density 1.7 g/cm^3^, tensile strength 3500 MPa, stiffness modulus 230 Gpa, and elongation at break 1.5% (Figure 4b).

Hybrid reinforcement of composites was used to obtain the optimal strength properties, i.e., compressive strength (steel fiber) and bending strength (carbon fibers). Fibers were added in an amount of 2%, 1.5%, 1.0%, and 0.5% by weight to fly ash and microspheres used as the matrix of the geopolymer mass.

### 2.2. Samples Preparation

**Figure 5:**
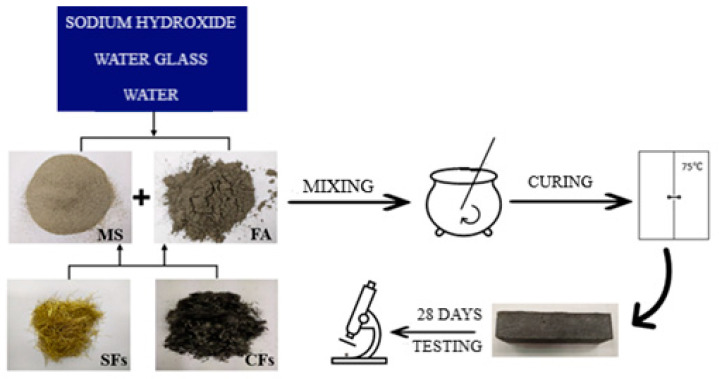
Scheme for the preparation of samples.

The samples were prepared according to the order presented in Figure 5.

First, a solution of 10 M NaOH (PCC Rokita SA, Brzeg Dolny, Poland), tap water, and water glass (STANLAB, Gliwice, Poland) was prepared-the ingredients were mixed and allowed to equilibrate to constant concentration and temperature before combining with the rest of the ingredients. Next, fly ash, microspheres, and/or steel and/or carbon fibers were weighed in appropriately selected proportions (Table 2).

The solid ingredients were preliminarily mixed for 2 min by using Varimixer Bear (Varimixer, Broendby, Denmark). After this time, the alkali solution was added and mixed next for 4 min. Then, the geopolymer mass was transferred to the previously prepared forms and compacted on a vibrating table. The molds with geopolymer mass were cured for 24 h at a temperature of 75 °C. Finally, the samples were cooled to ambient temperature, removed from the molds, and seasoned in the laboratory for 28 days.

### 2.3. Methods

Composite density was determined on the samples remaining after the strength test for compression using the geometric method. Firstly, the samples were measured with a caliper, and the volume was calculated from the dimensions obtained. All samples were then weighed on a laboratory scale. Finally, the obtained results were converted into the density according to Formula (1):

(1) $$\rho =\frac{m}{v}$$

where: ρ—density [g/cm^3^], m—mass [g], v—volume [cm^3^].

The compressive strength of the composites was carried out according to EN 12390-3: 2019-07: Concrete tests-part 3: Compressive strength. It was tested on the MATEST 3000 kN (Matest, Treviolo, Italy). Cubic samples of 50 × 50 × 50 mm were prepared for the test and seasoned for 28 days at room temperature. Each geopolymer composite was tested on 5 samples. The research was conducted in the following steps:

- The samples were cleaned, and loose debris was removed so that they did not come into contact with the clamping plates.
- The samples were placed in the testing machine so that they were in the center of the lower pressure plate.
- The load direction was perpendicular to the direction in which the samples were formed.
- A constant load speed of 0.5 N/mm^2^∙s was assumed.
- The load was increased continuously until the maximum value was reached.
- The compressive strength was determined by the machine program from Formula (2):
  (2) $$f_{c}=\frac{F}{A_{C}}$$
  where: $f_{cf}$—compressive strength [MPa], F—maximum load [N], $A_{C}$—the cross-sectional area of the sample [mm].

The bending strength of the composites was tested according to PN-EN 12390-5: 2019-08 standard: Concrete tests-part 5: Bending strength. It was made on the MATEST 3000 kN (Matest, Treviolo, Italy). Prismatic samples of 50 × 50 × 200 mm were prepared for the research and seasoned for 28 days at ambient temperature. Each geopolymer composite was tested on 5 samples. The study was carried out in the following steps:

- The samples were cleaned; loose debris was removed so that they would not come into contact with the rollers (the gap between rollers was 150 mm).
- The samples were placed in the testing machine so that they were properly centered and that their longitudinal axis was set at right angles to the longitudinal axis of both rollers.
- The load direction was perpendicular to the direction in which the samples were formed.
- A constant load speed of 0.05 N/mm^2^∙s was assumed.
- The load was increased continuously until the maximum value was reached.
- The bending strength was determined by the machine program from Formula (3):
  (3) $$f_{cf}=\frac{3\cdot F\cdot I}{2\cdot d_{1}\cdot d_{2}{}^{2}}$$
  where: $f_{cf}$—bending strength [MPa], F—maximum load [N], I—the spacing of the support rollers [mm], $d_{1},\;d_{2}$—transverse dimensions of the sample [mm].

Examination of the microstructure of composites was made on A JEOL JSM-820 scanning electron microscope (IXR Inc., Austin, TX, USA). For the research, the material remaining after the strength tests (compressive strength and bending strength test) was used. Firstly, the small amounts of the materials were dried to constant weight. Then they were placed on a carbon bed to drain the sample charge. Next, the samples were covered with a thin layer of gold using a JEOL JEE-4X sputtering machine. The observations were made at various magnifications between 50–2000×.

Research on thermal insulation properties was carried out using the NETZSCH HFM 446 device (NETZSCH-Gerätebau GmbH, Selb, Germany). Two kinds of samples were tested, with microspheres and the reference sample, and for each of them, four measurements were made. The dimensions and weights of the tested samples are presented in Table 3. 

## 3. Results and Discussion

This section may be divided into subheadings. It should provide a concise and precise description of the experimental results, their interpretation, as well as the experimental conclusions that can be drawn.

### 3.1. Density and Microstructure Research

**Figure 6:**
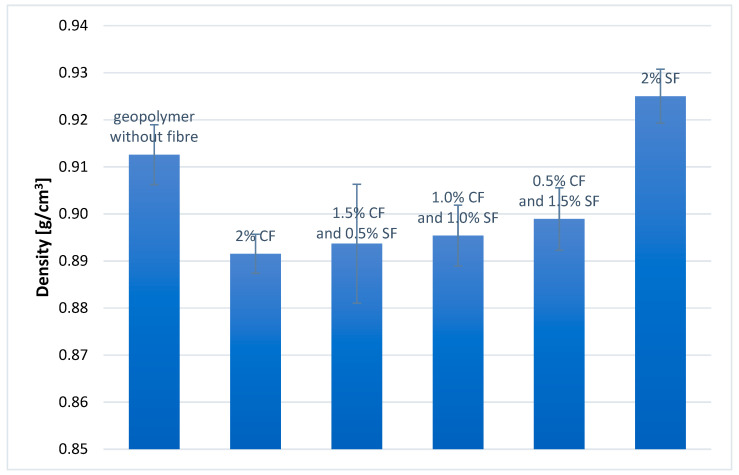
Density of geopolymer composites without/with fibers.

The density of the samples was determined using the geometric method. Each geopolymer composite was tested on five samples also used for the compressive strength test. Moreover, the standard deviation was calculated for each composite. The results are presented in Figure 6.

The density of all composites obtained is between 0.89 g/cm^3^ and 0.93 g/cm^3^. Density changes are not significant, taking into account fiber admixtures. This value is much lower than for the standard geopolymer created using sand as a fine aggregate, where the density value is usually between 1.3 and 1.80 g/cm^3^ [2,42]. Therefore, the composites with microspheres belong to the category of lightweight aggregate concretes. In addition, it can be seen that the density of geopolymer composites with fibers increases with the mass fraction of steel fibers. The highest density (~0.93 g/cm^3^) is characteristic for a geopolymer composite containing 2.0 wt.% SFs. The lowest density (~0.89 g/cm^3^) composite containing 2.0 wt.% CFs. The geopolymer composite without fibers has a density of ~0.91 g/cm^3^. On the other hand, the other composites containing CFs and SFs, in different proportions, have intermediate densities ranging from 0.89 to 0.90 g/cm^3^. It is worth noting that the density is decreed with the addition of CFs, even though they have a higher density than the pure matrix. It can be explained by additional voids in the materials created by fibers admixtures. 

The type of reinforcing fiber has a direct impact on the density of the composite. When the fiber density is lower than that of the geopolymer matrix, the bulk density of the geopolymer composite decreases. Conversely, when the fibers have a higher density than the matrix, the situation is quite different. The density of such a composite may increase, decrease, or remain at the same level as the density of the matrix itself. This is because differences in density caused by fiber admixtures are not the only rule governing the entire process. For example, the emerging porosity may appear in the structure of the composite as a result of retaining air bubbles in the structure of the composite, which reduces its density.

**Figure 7:**
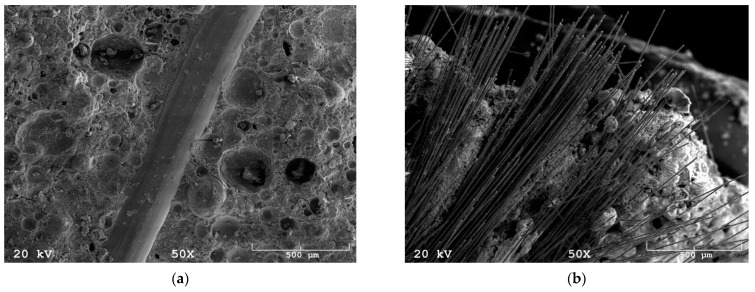
(**a**) SEM image: steel fiber in geopolymer matrix, magnification 50×; (**b**) SEM image: carbon fibers in geopolymer matrix, magnification 50×.

Examination of the steel and carbon fibers shows that they are characterized by a smooth surface and constant dimensions throughout their length. The breakthrough observations show that the fibers are coherent with the geopolymer matrix (Figure 7a). The carbon fibers appear to create agglomeration in the geopolymer matrix (Figure 7b), but it is connected with their internal construction, where each carbon fiber is made of many tiny strands of fiber joined together. This feature of CFs could explain the changes in density.

Microstructural research shows a typical morphology for reinforced geopolymers with fibers [43,44,45]. Short fibers are regularly dispersed in all volumes of the matrix and are consistent with them.

### 3.2. Compressive Strength

**Figure 8:**
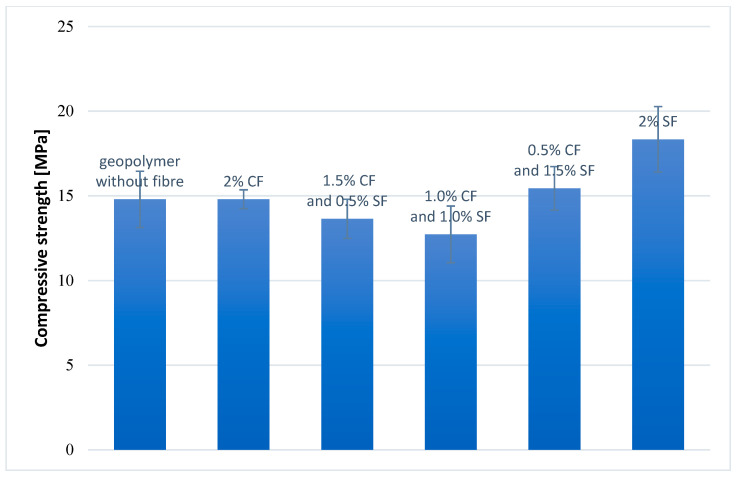
Compressive strength of geopolymer composites.

The compressive strength test was carried out on cubic samples 50 × 50 × 50 mm, seasoned at room temperature for 28 days. Each geopolymer composite was tested on five samples. Moreover, the standard deviation was calculated for each composite. The results are presented in Figure 8.

The best compressive strength is obtained for a geopolymer composite containing 2 wt.% SFs (18.3 MPa). In turn, the lowest is for a composite containing 1.0 wt.% CFs and 1.0 wt.% SFs (12.7 MPa). The fiber-free composite has a compressive strength of 14.8 MPa, which is a value practically the same as the compressive strength of the composite containing 2.0 wt.% CFs (14.8 MPa) and a composite containing 0.5 wt.% CFs and 1.5 wt.% SFs (15.4 MPa). For the composite with 1.5 wt.% CFs and 0.5 wt.% SFs have a compressive strength of 13.6 MPa. 

The results show that only the addition of steel fibers in an amount of 2 wt.% significantly increases the compressive strength of geopolymer composites (about 24%). For other composites, the changes are slighter. A small increase of compressive strength value was observed for a composite containing 0.5 wt.% of CFs and 1.5 wt.% of SFs (about 4%). The value of compressive strength was on the same level for a composite containing 2 wt.% CFs and lower by about 8% for a composite containing 1.5 wt.% CFs and 0.5 wt.% SFs and about 14 wt.% for a composite comprising 1.0 wt.% CFs and 1.0 wt.% SFs. The obtained results can be due to the lack of coherence between the fibers and the matrix in the case of hybrid reinforcement of composites. In addition, a scattering of the results between the samples is observed, which may indicate heterogeneity in the structure of the geopolymeric mass.

### 3.3. Bending Strength

**Figure 9:**
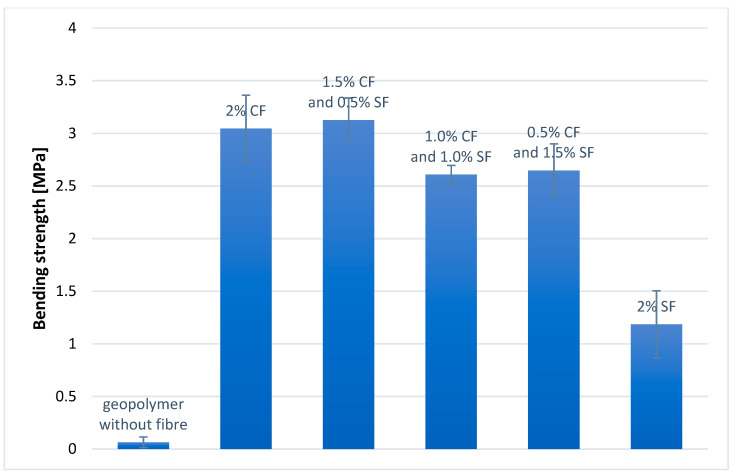
Bending strength of geopolymer composites.

The bending strength test was carried out on 50 × 50 × 200 mm prismatic samples, seasoned at ambient temperature for 28 days. Each geopolymer composite was tested on five samples. Moreover, the standard deviation was calculated for each composite. The results are presented in Figure 9.

The best result of the bending strength was obtained for a geopolymer composite containing 1.5 wt.% CFs and 0.5 wt.% SFs (3.13 MPa). In turn, the lowest for composite without fibers (0.064 MPa). The composite containing 2 wt.% SFs has a bending strength of 3.05 MPa, which is a value slightly lower than the bending strength of the composite containing 1.5 wt.% CFs and 0.5 wt.% SFs. The composite containing 2.0 wt.% SFs has the lowest strength—1.19 MPa of all fiber-reinforced composites. The composites containing 1.0 wt.% CFs and 1.0 wt.% SFs as well as 0.5 wt.% CFs and 1.5 wt.% SFs have bending strengths of 2.61 MPa and 2.65 MPa, respectively. The results show that the addition of reinforcement fibers to the geopolymer composite with a fly ash matrix and microspheres significantly improves the bending strength in all cases.

**Figure 10:**
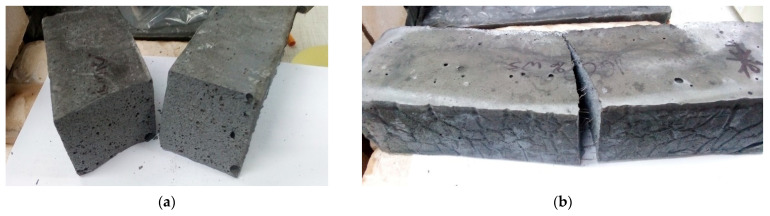
(**a**) An example photo showing the breakthrough of a geopolymeric composite without fibers; (**b**) An example photo showing the breakthrough of a geopolymeric composite reinforced with steel fibers.

Furthermore, the exemplary breakthroughs are presented in Figure 10a,b.

### 3.4. Thermal Conductivity of Lightweight Matrix

Table 4 shows the density (ρ), thermal conductivity (λ), and compressive strength (Rs) of geopolymer composites consisting of 50 wt.% fly ash and 50 wt.% sand/microspheres.

Geopolymer composite containing 50 wt.% fly ash and 50 wt.% sand, similarly to the composite in which sand is replaced with microspheres, is characterized by a low thermal conductivity coefficient. A composite containing a matrix consisting in part of microspheres is also characterized by a very low density, lower than that of a composite made of sand. In addition, the traditional geopolymer composite with sand has a much higher compressive strength than a composite in which a fine-grained aggregate has been replaced with light material-microspheres.

## 4. Discussion

The hybrid reinforcement of lightweight geopolymers is very limited [2,7,12]. Additionally, in the literature, there is a lack of investigation for geopolymers materials reinforced by carbon and steel fibers. Due to this, the discussion and comparison-obtained results were selected articles that describe investigations focused on hybrid reinforcement with steel as one component and other fiber as a second component [46,47]. The research is different combinations with polymer fibers [2,7,12,46,47].

The results obtained for compressive strength are in line with other research on hybrid reinforcement of materials, including steel fibers combined with plastic fibers [2,7,48,49]. An example can be tests carried out on a geopolymer material with a matrix made of fly ash and smoke silica, reinforced with hybrid hooked steel fiber (length: 60 mm) and PP fiber (length: 58 mm) [48]. Two series of fiber-containing samples were prepared. In the first series, PP fibers were replaced with steel fibers with an increment of 0.2% until completely replaced. In the second one, steel fibers were added to the composite with the same increment until it reached 2% by volume. In both cases, the test was carried out after 28 days of seasoning, and an increase in compressive strength was observed with the increase in the number of steel fibers in the composite. In the first experiment, it increased from 40.50 MPa (for 0.8% PP and 0.2% SFs) to 60.57 MPa (for 1.0% SFs). However, in the second study, it increased from 39.40 MPa (for 0.8% PP and 0.2% SFs) to 72.99 MPa (for 1.0% PP and 1.0% SFs) [48].

Hybrid reinforcement of the material in the form of corrugated steel fibers (length: 25 mm, diameter: 0.55 mm) and hook-shaped (length: 25 mm, diameter: 0.3 mm) and PE fiber (length: 12 mm, diameter: 0.012 mm) was also used on a geopolymer matrix made of fly ash and slag [49]. The samples were made of: the matrix material itself, containing 2.0% by volume of fibers (1.0% each of crimped and hooked SFs; 0.9% of crimped and hooked SFs each, and 0.2% of PE; 0.8% of crimped each) and hook-shaped and 0.4% PE), containing 1.5% by volume of crimped and hooked SFs (0.75% each of crimped and hooked SFs), containing 1.0% by volume of fibers (0.5% of crimped and hooked SFs each; 0.4% notched and hooked SFs and 0.2% PE; 0.8% notched and 0.2% PE). The test was carried out after 28 days of storage. The highest compression strength was observed for composites reinforced with only steel fibers. The samples reinforced with the same volume of mixed fibers were characterized by reduced compressive strength. The best compressive strength equal to 82.05 MPa was obtained for the composite containing 2.0% by volume of steel fibers. In turn, hybrid reinforced fibers (SFs and PE) were characterized by compressive strength (68.88 MPa–72.87 MPa) analogous or even lower than the strength obtained for the matrix material alone (72.01 MPa) [49].

Hybrid reinforcement in the form of hooked steel fibers (length 51 mm) and melamine (length 5 mm) was used in the case of a geopolymer composite with a matrix made of fly ash and sand. Samples containing 1.0 wt. % fibers in the following ratio of carbon fibers to melamine fibers: (50: 0, 100: 0, 0:50, 0: 100, 20:80, 40:80, 60:40, 80:20) and consisting of the matrix material itself [2]. The compressive strength results showed that with one type of fiber reinforcement, better results are obtained with the addition of 0.5 wt.% fibers than 1.0 wt.% Higher compressive strengths were obtained for the reinforced composite with melamine fibers. In the case of hybrid composite reinforcement, a synergistic effect was observed. The highest compressive strength values were obtained for the composite containing 0.6 wt.% melamine fibers and 0.4 wt.% SFs (47.2 MPa) and containing 0.3 wt.% melamine fibers and 0.8 wt.% SFs (47.8 MPa) [2].

The results obtained for the bending strength are consistent with other research work [2,46,47,48]. The study provided by Jia et al. [50] was carried out on geopolymer composites with a 40% volume fraction of microspheres, the flexural strength of the composite reached 5.6 MPa, while for the matrix material consisting of metakaolin alone, it was 28.5 MPa. Furthermore, the strength properties of the composite decreased with the increasing volume fraction of microspheres [50]. Thus, it is observed that microspheres can significantly reduce the strength properties of geopolymer composites. 

In comparison to other studies provided for geopolymer composites with hybrids reinforced, especially with using steel and polymer fibers, the following remarks can be drawn:

- In a study carried out on a geopolymer material with a matrix made of fly ash and smoke silica-reinforced with hybrid hooked steel fiber and polypropylene (PP) fiber, the same relationship was observed for the bending strength as for compressive strength. With the increasing content of steel fibers, the value of the bending strength of composites increases [48].
- In the study of a geopolymer composite with a matrix made of fly ash and slag reinforced with hybrid fibers corrugated and hooked steel fibers and PE fiber, it was observed that the strength properties of the hybrid reinforced composites were higher in the bending strength test. In the composite without the addition of fibers, the bending strength reached the lowest value of 3.89 MPa. In turn, the highest value of 11.3 MPa was achieved in a composite containing 0.8% by volume of crimped and hooked fibers and 0.4% by volume of PE [49].
- In a study conducted on a geopolymer composite with a matrix made of fly ash and sand reinforced with hybrid hooked steel and melamine fibers, the results of bending strength showed that he addition of melamine fibers as well as steel fibers increased the flexural strength in comparison to the plain matrix. The best results were obtained for the combination of both types of fibers but not exceeding the maximum value of 1% by weight [2].

The values of the properties obtained for the thermal conductivity of the geopolymer matrix can be compared with the traditional materials used in construction (Table 5).

Thermal insulation materials play an essential role in construction. A well-insulated building means, above all, lower heating costs, but also a more favorable microclimate of the rooms [52,53]. The production of new insulation materials makes it possible to reduce the energy consumption of buildings without reducing the comfort of their users. The exterior walls are one of the most important structural elements of buildings. Their task is to transfer loads to the foundation benches or provide thermal insulation [51,52]. The functionality of the outer wall depends on the masonry elements used, the mortar (masonry and the plastering), and insulation. When using reinforced concrete, silicate brick, or ceramic brick as a building material, the building should be insulated, e.g., with polystyrene or mineral wool. However, such action is not necessary when building from low-density aerated concrete blocks with a low thermal conductivity coefficient. Although the range of compressive strength of aerated concrete is not wide, it can be used successfully to erect load-bearing walls as well in multi-story buildings [52,53]. Geopolymer matrix containing 50 wt.% FA and 50 wt.% MS is characterized by a thermal conductivity coefficient close to that of aerated concrete. Moreover, this material has a higher compressive strength than it.

## 5. Conclusions

An important direction of development of modern civil engineering is therefore looking for new, more ecological materials, in particular those based on renewable raw materials. One of the promising alternatives may be geopolymerization technologies with a much lower carbon footprint compared to traditional building materials. In addition, the production of lightweight geopolymer materials can effectively improve the thermal insulation properties of buildings, reduce transportation and assembly costs, and reduce the load-bearing capacity of the structure. Additionally, the use of microspheres for the production of geopolymers enables the protection of a very valuable fine-grained aggregate used in the production of concrete sand.

By analyzing the results of the research presented in this paper, the following conclusions can be drawn:

- All the obtained composites have densities in the range of approx. 0.89–0.93 g/cm^3^. Therefore, they can be classified as lightweight aggregate concretes.
- The best compressive strength is obtained for a steel fiber (2.0% by weight).
- In all cases, the addition of reinforcing fibers to lightweight geopolymer composites significantly improves the bending strength value and may change the fractured nature of the brittle material to a more ductile one. The best bending strength is obtained for the hybrid reinforced composite: 1.5 wt.% CFs and 0.5 wt.% SFs.
- Analysis of the microstructure of the steel and carbon fiber-reinforced composite shows good coherence of the fibers with the matrix.
- The geopolymer composite is characterized by low thermal conductivity (0.22 W/m K) at low density, giving values comparable to aerated concrete blocks. Geopolymer matrix is made of fly ash and microspheres and is therefore characterized by better thermal insulation properties (0.18 W/m K).

Light geopolymer composites with a matrix made of lightweight aggregates-fly ash and microspheres have great potential for use in construction. However, further tests are necessary, in particular determining their resistance to changing environmental conditions and confirming the stability of material properties in the long term.

## Figures and Tables

**Table 1:** Oxygen composition of FA.

Chemical Formula	%	Chemical Formula	Concentration %
**SiO_2_**	52.076	**BaO**	0.060
**Al_2_O_3_**	28.697	**SrO**	0.053
**Fe_2_O_3_**	5.767	**Cl**	0.048
**CaO**	3.467	**ZrO_2_**	0.028
**K_2_O**	2.424	**ZnO**	0.024
**Na_2_O**	2.384	**CuO**	0.023
**MgO**	1.997	**Cr_2_O_3_**	0.020
**SO_3_**	1.220	**Co_3_O_4_**	0.018
**TiO_2_**	1.107	**PbO**	0.017
**P_2_O_5_**	0.475	**NiO**	0.017
**MnO**	0.060	**Rb_2_O**	0.016

**Table 2:** Samples composition.

No.	FA [g]	MS [g]	Alkali Solution [mL]	SFs [g]	CFs [g]
1	980	980	900	0	0
2	980	980	900	0	40
3	980	980	900	10	30
4	980	980	900	20	20
5	980	980	900	30	10
6	980	980	900	40	0

**Table 3:** Dimensions and weight of samples for testing thermal insulation properties.

Sample	Dimensions [mm]	Weight [kg]
Geopolymer (50 wt.% FA + 50 wt.% sand)	137.6 × 138.2 × 26.2	0.428
Geopolymer (50 wt.% FA + 50 wt.% MS)	137.3 × 138.4 × 25.5	0.648

**Table 4:** Selected properties of geopolymer matrices.

Sample	ρ [kg/m^3^]	λ [W/m·K]	R_s_ [MPa]
Geopolymer (50 wt.% FA + 50 wt.% sand)	1339	0.22	42.0
Geopolymer (50 wt.% FA + 50 wt.% MS)	859	0.18	14.8

**Table 5:** Properties of selected building materials. Part of data from Ref. [51].

Sample	ρ [kg/m^3^]	λ [W/m·K]	R_s_ [MPa]
Silicate brick	900–2200	1.10	7.5–15.0
Ceramic brick	1800	0.77	5.0–35.0 and more
Aerated concrete block	300–1000	0.20	2.0–7.5
Reinforced concrete	2400	1.80	20–150 and more
Mineral wool	10–200	0.055	−
Styrofoam	10–50	0.040	−
Wood	550	0.20	−
Lime plaster	1700	0.80	0.3–4.0
Cement-lime plaster	1850	0.90	1–20
Cement plaster	2000	1.20	1–30
Plasterboard	1000	0.9	−
Geopolymer (50 wt.% FA + 50 wt.% sand)	1339	0.22	42.0
Geopolymer (50 wt.% FA + 50 wt.% MS)	859	0.18	14.8

## Data Availability

Not applicable.
